# From research to application: evaluation of literature-based and newly identified GWAS and GP-derived loci for anthracnose resistance in white lupin, across validation panels and environments

**DOI:** 10.1007/s11032-026-01661-w

**Published:** 2026-04-22

**Authors:** András Patyi, Michael Schneider, Christine Arncken, Michał Książkiewicz, Monika M. Messmer, Grit Schwertfirm, Mariateresa Lazzaro

**Affiliations:** 1 Department of Crop Sciences, Research Institute of Organic Agriculture (FiBL), Ackerstrasse 113, Frick, 5070 Switzerland; 2 Institute of Plant Genetics, Polish Academy of Sciences, Strzeszyńska 34, Poznań, 60-479 Poland; 3 Institute for Crop Science and Plant Breeding, Bavarian State Research Center for Agriculture (LfL), Am Gereuth 2, Freising, 85354 Germany

**Keywords:** White lupin, Anthracnose, Marker assisted selection (MAS), Genome-wide association study (GWAS), Genomic prediction (GP), Molecular breeding

## Abstract

Resistance to anthracnose disease, caused by *Colletotrichum lupini*, in white lupin is polygenic and rare in global germplasm, making breeding for resistance challenging. Several studies have identified genomic regions associated with resistance, but reported QTLs rarely overlap. Overall, anthracnose-associated loci have not yet been appropriately exploited in breeding due to the lack of validation, especially in unrelated material. We assessed three literature-based SNP-markers associated to a previously described QTL, as well as six originating from two published GWAS studies. In addition, by re-analysing two published datasets, we newly (i) identified five SNP-markers through a genome-wide association study (GWAS) and (ii) assembled a genomic prediction (GP) model based on a set of 42 SNP-markers. All SNP-markers were transformed to PCR-based assays to facilitate cost-efficient application in breeding and tested in a diversity panel and two breeding panels, phenotyped in field and/or controlled conditions. We validated the association to anthracnose resistance for the previously reported QTL on chromosome *Lalb_Chr10*, tagging a source of resistance in the resistant cultivar ‘Frieda’. Two markers showed significant associations (*p* < 0.05) with anthracnose resistance in at least two panels, and an additional seven markers were significant in one. The GP model had a prediction accuracy of 0.84 (± 0.13) in the training panel, but is not applicable as such in the unrelated validation panels. However, re-training the GP model with the respective validation panel data showed clear prediction improvement buffering the limitation of environmental context and narrower genepools applied in a breeding programme.

**Supplementary Information:**

The online version contains supplementary material available at 10.1007/s11032-026-01661-w.

## Introduction

White lupin (*Lupinus albus* L.; diploid, 2n = 50) is a Mediterranean grain legume with high potential as a protein source for feed and food (Hufnagel et al. 2020). However, its cultivation is severely constrained by anthracnose, caused by the ascomycete *Colletotrichum lupini*. All lupin species are susceptible to this disease, which is particularly destructive in temperate regions of Europe. Anthracnose infects all aerial plant parts, impairing vegetative growth and pod development, and can result in complete yield loss in unfavourable years (Alkemade et al. 2025).

The major outbreak of this fungal pathogen, with suggested centre of origin in Latin America (Alkemade et al. 2021a, b, 2023), dates in the 1970 s and 80 s in Europe and South America. It quickly became a worldwide pandemic destroying lupin fields, especially since the commonly used commercial varieties at the time showed to be susceptible to this disease (Talhinhas et al. 2016). Thus, the new objective of plant breeders was to develop resistant varieties against *C. lupini*. For white lupin, a large screening effort was conducted in Australia between 1997 and 1998, assessing anthracnose resistance in ca. 300 genetic resources of white lupin from across the globe (Adhikari et al. 2009, 2013). The only identified source of resistance was found in an Ethiopian genepool of landraces collected in the highland region (1900–2600 m altitude) in the provinces of Gonder and Gojam. The most resistant accessions were collected from the Eastern Gojam region (Debre Markos district), including accessions P27174, P27175, and P27178 (Adhikari et al. 2009). More recently, a large number of genetic resources (gene bank accessions, landraces, cultivars and breeding lines) were evaluated in the field by FiBL (approximately 100 accessions per year from 2015 to 2022, 890 in total; unpublished data). Accessions were screened once late in the growing season (BBCH82-87) for their disease scores ranging from 1 to 9 (1 – healthy, 9 – dead, as described by Alkemade et al. 2021a, b), grown with an infection row on each side. No accessions showed complete resistance against the pathogen, and field performance further confirmed the rarity of increased resistance in the global white lupin germplasm.

Motivated by the severe impact of anthracnose on white lupin cultivation, numerous molecular studies have investigated the genetic basis of resistance. Early work focused on a bi-parental F_8_ recombinant inbred line (RIL) population derived from the cross `Kiev mutant` (susceptible, CPVO, 2026)× P27174 (resistant, Adhikari et al. 2009). Using this population, Phan et al. (2007) identified two major quantitative trait loci (QTLs) on linkage groups LG04 and LG17, corresponding to current chromosomes *Lalb_Chr04* and *Lalb_Chr02*, which together explained more than half of the phenotypic variance (31% and 26% respectively). These results suggested that anthracnose resistance is a quantitative trait under polygenic control. Subsequent analyses of the same RIL population refined this genetic framework and proved the trait`s polygenic nature. Yang et al. (2010) mapped three resistance-associated markers (WANR1 and WANR2 on Lalb_Chr02, and WANR3 on *Lalb_Chr04*) together explaining 59% of phenotypic variation. The development of a high-density consensus linkage map (Książkiewicz et al. 2017) further confirmed QTLs on *Lalb_Chr02* and *Lalb_Chr04* and identified an additional locus on *Lalb_Chr10*. Integrating these findings, Rychel-Bielska et al. (2020) evaluated the available markers for breeding applications and developed three CAPS markers suitable for marker-assisted selection (MAS) targeting the QTLs on *Lalb_Chr02* and *Lalb_Chr04*, while reporting limited success of the WANR markers in breeding programs. Despite these advances, the transferability of resistance-associated markers remained a challenge. A genomic prediction model developed by Alkemade et al. (2021a, b), based on the markers reported by Rychel-Bielska et al. (2020), showed no correlation between predicted and observed resistance values. Notably, the resistant parental line of the RIL population (P27174) exhibited an opposite anthracnose resistance pattern in the 2021 study (Alkemade et al. 2021a, b) compared to that displayed in their original phenotyping in Australia (Adhikari et al. 2009), underscoring the strong influence of environment and experimental conditions.

The availability of a reference genome for the cultivar Amiga (CPVO, 2026), together with resequencing data from 39 accessions (Hufnagel et al. 2020, 2021), enabled a shift from bi-parental mapping to genome-wide association studies (GWAS) in more diverse germplasm. Alkemade et al. (2022) analysed a panel of 181 accessions using 4,611 SNPs and phenotyping with stem-wound inoculation method in controlled conditions. This GWAS identified three closely linked SNPs on *Lalb_Chr05* associated with anthracnose resistance and reported additional resistant accessions, including material from Ethiopia, two Chilean breeding lines, and the newly released cultivar `Frieda` (CPVO, 2026).

However, the robustness of these associations was challenged by later work. Schwertfirm et al. (2024) conducted GWAS and genomic prediction using 24,576 SNPs in a panel of 255 accessions phenotyped across multiple years, locations, and developmental stages under field condition. This study could not validate the *Lalb_Chr05* associations, nor the resistance of the previously reported Chilean accession, a result corroborated by independent field trials in Switzerland (FiBL, unpublished data). Instead, Schwertfirm et al. (2024) identified 17 significant SNPs distributed across 14 chromosomes. Only three of these overlapped with loci previously reported in the `Kiev mutant` × P27174 population, corresponding to known QTLs (*Lalb_Chr04*, *Lalb_Chr10*).

Collectively, these studies highlight the major challenges facing anthracnose resistance breeding in white lupin. Resistance is scarce in global germplasm, strongly influenced by phenotyping methodology and environment, and controlled by multiple loci with limited consistency across studies. The poor overlap between reported QTLs and GWAS signals has hindered the effective and consistent deployment of marker-assisted selection. Consequently, a key remaining challenge is the validation of cost-efficient molecular assays that reliably tag the respective resistance loci across independent gene pools, with particular emphasis on breeding-relevant material. Addressing this gap is essential for translating molecular discoveries into practical resistance breeding strategies.

The present study aims to address this research to application gap by (i) exploiting already published datasets for further loci identification with GWAS, and for developing a GP model based on a minimal number of markers as a blue-print for low cost application; (ii) merging all suitable literature and newly identified markers to one marker assay-type, PCR Allelic Competitive Extension (PACE), tagging reference and alternative alleles with distinct fluorescent signals; (iii) testing these SNP-markers in new genetic pools (namely one diversity panel and two breeding panels) and against different phenotyping approaches (field and controlled conditions); (iv) applying a GP model retraining approach to improve performance in the selection of breeding materials.

Overall, this study develops and evaluates SNP marker assays for application in anthracnose resistance selection, based on the hypothesis that switching from marker-trait discovery to application can be limited by (i) a different, and in some cases narrower genepool and (ii) the origin of the phenotypic approach in the discovery phase compared to the validation.

## Materials and methods

### Plant material

#### Public datasets

The LUW (LUpin White, *n* = 255) panel (downloaded from the supplementary material of Schwertfirm et al. 2024), consists of 167 gene bank accessions, 73 advanced breeding lines from the Landwirtschaftliche Lehranstalten Triesdorf (LLT, Germany) and 15 commercially available cultivars (Table 1).

**Table 1:** Description of the panels used, including panel name, type, material composition, number of individuals and the phenotyping environment(s) with information on the phenological stage of the plants

Panel	Type	Use	Materials	*n*	Phenotyping
LUW	Public	New GWAS	landraces breeding lines cultivars	255	field adult (3 phenological stages) (Schwertfirm et al. 2024)
Training	Public	New GP	landraces breeding lines cultivars	192	climate chamber seedling (2–4 weeks-old) (Alkemade et al. 2022)
Diversity	New	Marker validation	wild type landraces breeding lines cultivars	48	climate chamber seedling (2–4 weeks-old) experiment in 2022
Multi-parental	New	Marker validation	breeding lines (F_4_-F_6_)	20 + 2^a^	climate chamber seedling (2–4 weeks-old) experiment in 2024
				94 + 2^a^	field adult (3 scorings) experiment in 2024
Biparental	New	Marker validation	breeding population (F_3)_	50	field adult (1 late-season scoring) experiment in 2024

^a^ The first number indicated the size of the validation panel, the second represents the cultivars `Frieda` and `Amiga`, which were added to the validation populations for reference

The panel from Alkemade et al. 2022 (downloaded from the supplementary material of the study) consists of 200 commercially available cultivars, landraces and breeding lines (training panel) with enough phenotypic data available for 192 of them (Table 1).

### New diversity and breeding panels

The genetic pools (Table 1) newly phenotyped in this study to validate the consistency of individual marker effects and the accuracy of genomic prediction are (i) a diversity panel, curated with the intention to represent diverse genetic backgrounds and diverse sources of resistance, phenotyped in controlled conditions; (ii) a multi-parental panel of F_4_-F_6_ breeding lines, phenotyped in controlled and field conditions; (iii) a biparental panel consisting of F_3_ individuals with low quinolizidine alkaloid content. The latter two are breeding panels from the common FiBL and gzpk (*Getreidezüchtung Peter Kunz*, Feldbach, Switzerland) breeding programme (Table 1).

The **diversity panel** includes 48 accessions consisting of 20 breeding lines, 23 commercial cultivars, 4 landraces and the wild type *graecus* (Table 1, Supplementary Table S1). The **multi-parental panel** consists of 94 individuals, selected from 25 advanced breeding lines (3–5 individual plants of each, generation from F_4_ to F_6_), and two commercial cultivars used as controls for resistance (`Frieda`) and susceptibility (`Amiga`). All single plants were pheno- and genotyped individually, thus the entire panel represented 96 individuals. Their simplified pedigree, generation, disease score and allelic states are presented in Supplementary Table S2. All 94 individual plants from the 25 breeding lines were analysed in field experiments, whereas in the controlled environment, one representative on only 20 of these lines were analysed due to limited seed availability (by sowing 6 seeds from each plant) (Supplementary Table S3).

The **biparental panel** consisted of 50 individuals (Table 1) in F_3_ generation, originating from an elite cross between cultivars `Frieda` x `Dieta` (Supplementary Table S4, CPVO, 2026) exhibiting exceptionally low quinolizidine alkaloid content through two independent loci associated low-alkaloid chemotype (Patyi et al. 2025). Segregating for resistance to anthracnose, this population served the purpose of the application of these molecular markers for selection of individuals with increased resistance.

### New phenotypic scoring and analysis

Experiments in field conditions were conducted for the multi-parental and biparental panels in 2024 (Table 1, Supplementary Table S2, Supplementary Table S4), whereas in controlled conditions for diversity panel in 2022 (Table 1, Supplementary Table S1) and multi-parental panel in 2024 (Table 1, Supplementary Table S3).

In field experiments, each breeding line in the multi-parental panel was grown in unreplicated rows consisting of six individuals, surrounded by infection rows on both sides. The commercial cultivars `Frieda` and `Amiga` were repeated three times across the trial. The individuals of the biparental panel were grown without infection rows in the same field with high anthracnose infection pressure. Individuals of both breeding panels were grown under protective nets to prevent cross-pollination in Full-Reuenthal, Switzerland (47°35’59.5"N 8°12’10.4"E) in 2024. Phenotypic scores of the multi-parental (Supplementary Table S2) and the biparental panel (Supplementary Table S4) from the field correspond to scores of individual plants collected during the growing season. For the multi-parental panel, individuals were scored three times across the growing season (102, 120 and 128 days after sowing) to calculate standard area under disease progression curve (sAUDPC) scores (calculated using the package *agricolae* version 1.3–7.3 (Mendiburu 2023) in R version 4.4.2 (R Core Team, 2024), while the representatives of the biparental panel were scored only once in the field (BBCH82-87), to represent a less labour-intensive and more realistic phenotyping approach for early generation material.

In controlled environment experiments, artificial inoculation was performed as presented in Alkemade et al. (2021a, b) using the highly virulent *C. lupini* JA01 strain, isolated from Mellikon, Switzerland, in 2018. One individual plant representative for each breeding line in the multi-parental panel was selected, and 6 seeds of this plant were grown and phenotyped in the climate chamber. Each plant was assessed for disease symptoms at 0, 3, 7, 10 and 14 days post-inoculation to calculate sAUDPC scores (calculated using the package *agricolae* version 1.3–7.3 (Mendiburu 2023) in R version 4.4.2 (R Core Team, 2024). The adjusted sAUDPC scores of the offspring of the selected plants were correlated (Pearson correlation) to the sAUDPC values of the mother plants observed in the field.

The disease score for phenotypic values originating from controlled conditions was analysed using Linear Mixed Models (LMMs), with sAUDPC score as response variable, corresponding accession as fixed, and individual plant replication (*n* = 6) nested within the climate chamber experiment as a random effect for the re-analysis of the training panel and the phenotypic values for all retrained models with phenotypic values originating from independent climate chamber experiments, and only biological replication (*n* = 6) for the diversity- and multi-parental panels where phenotypic values originated from a single climate chamber experiment (Supplementary Table S1, Supplementary Table S3).

Estimated marginal means from the LMMs were calculated using *emmeans* version 1.11.1 (Lenth et al. 2025), which values were used for downstream analysis. Meanwhile, for the field observations, individual disease scores were used for the downstream analysis as individual plants were genotyped and phenotyped.

Broad sense heritability (H^2^) in the validation panels as described by Schmidt et al. (2019) using the package inti version 0.6.9.1 (R Core Team, 2024). It was not possible to calculate H^2^ values (Cullis) for the field datasets (multi-parental and biparental panels field observations) due to the unavailability of biological replication.

## SNP markers

### PACE assay design and cycling conditions

All loci from literature, as well as the newly identified ones had associated SNP loci, with known information of the location and available genomic context sequence on the white lupin genome browser (Hufnagel et al. 2020, 2021). DNA extraction was performed using Qiagen FTA cards (QIAGEN GmbH; Hilden, Germany). Primer triplets were designed by 3CR Bioscience (Essex, UK) based on the provided context sequence (± 100 bp) for each polymorphic locus, with FAM and HEX fluorophores tagging the reference and alternative allele, respectively. For each PACE assay, the sequences of the two forward and the common reverse primers are presented in Supplementary Table S5 (markers that were used individually for the evaluation), and in Supplementary Table S6 (markers used for GP model evaluation). PCRs were performed using the newly developed primer triplets, with cycling conditions as described in Patyi et al. (2025) ran on the CFX Opus 384 Real-Time PCR System (Bio-Rad Laboratories, Inc.; Hercules CA, USA). Allelic calls were assigned automatically using CFX Maestro version 2.2 (Bio-Rad Laboratories, Inc.; Hercules CA, USA) which were used for further analysis.

### Individually assessed SNP markers

The nine markers from literature include: (i) three from Rychel-Bielska et al. 2020, (ii) one from Alkemade et al. 2022 and (iii) five from Schwertfirm et al. (2024).

CAPS markers from Rychel-Bielska et al. (2020) tagging the QTLs (*Lalb_Chr02*, *Lalb_Chr04*) identified by Yang et al. (2010) were transformed as PACE marker assays. The context sequences of the three CAPS markers were downloaded on the respective chromosomes based on amplicon sequences (amplicon ID: TP222136, TP47110, TP338761; sequence retrieved from DDBJ, Altschul et al. 1997). In all three cases, the amplicon-associated sequence contained a SNP, which polymorphism was used for marker development.

Alkemade et al. 2022 identified three SNPs associated to anthracnose resistance. One out of the three, Lalb_Chr05_3706534 has only been associated to relative lesion size, which phenotypic measurement was not carried out in this study, therefore it was not considered for PACE assay development. The remaining two, Lalb_Chr05_2957601 and Lalb_Chr05_2957940 are closely linked (ca. 300 bp apart) being positioned on the coding sequence of the same gene on chromosome *Lalb_Chr05*. For this reason, in our study we used only SNP Lalb_Chr05_2957601.

Schwertfirm et al. (2024) report 17 SNPs associated to anthracnose resistance, from which the seven high confidence loci were considered for marker development. For Lalb_Chr19_2669672, PACE primer design was unfeasible due to the presence of other SNPs in the 50 bp flanking regions, while the SNP-marker Lalb_Chr04_16717968 failed during the PCR amplification. Thus, we used only five markers published by Schwertfirm et al. (2024) for evaluation in this study.

Furthermore, through the re-analysis of the datasets presented in Schwertfirm et al. (2024), we present two marker loci that overlap with the ones published in the original study, and present five additional markers, derived from the new GWAS analysis (Table 2, Supplementary Table S5). To complement the MMLM, FarmCPU and BLINK models presented in Schwertfirm et al. (2024), we performed the GWAS (raw data as provided in the original study) in R version 4.2.1. (R Core Team, 2022) using GAPIT3.3 (Wang and Zhang 2021) GLM function, including all markers, regardless of MAF and missing rate, to determine additional potential candidate loci. Including missing markers should enable the detection of “hidden” QTLs, as described by Gabur et al. (2018). All potential candidate SNPs have been assessed for relevance in the validation panels with PACE markers to account for the increased false positive detection rate of the GLM model.

**Table 2:** SNP-markers applied in this study for individual assessment. Locus of the target SNP on the respective chromosome, its status indicates if a specific SNP is newly described in this study or previously published, and the method with which it was identified. For de novo markers, p-values and explained variance is presented from the repeated GWAS analysis, specifying which model(s) describe the marker and which phenological stage`s phenotypic data (accessed from Schwertfirm et al. 2024) is it associated to, and how many accessions were genotyped (n)

Chromosome	Position	Dataset	Status^a^	Method	Phenotyping	*p*-value	Explained Variance (%)	*n*	Model
*Lalb_Chr02*	550,648	Rychel-Bielska et al. 2020	published	QTL mapping	field				
*Lalb_Chr02*	635,857	Rychel-Bielska et al. 2020	published		field				
*Lalb_Chr04*	16,724,499	Rychel-Bielska et al. 2020	published		field				
*Lalb_Chr05*	2,957,601	Alkemade et al. 2022	published	GWAS	controlled conditions				
*Lalb_Chr18*	12,358,001	Schwertfirm et al. 2024	published	GWAS	field				
*Lalb_Chr21*	3,907,702	Schwertfirm et al. 2024	published		field				
*Lalb_Chr24*	394,166	Schwertfirm et al. 2024	published		field				
*Lalb_Chr04*	2,315,230	Schwertfirm et al. 2024	*de novo*/published		field	1.39 × 10^− 6^	32	251/255	GLM
*Lalb_Chr10*	16,065,293	Schwertfirm et al. 2024	*de novo*/published		field	2.97 × 10^− 10^	61	255/255	GLM
*Lalb_Chr06*	2,629,006	Schwertfirm et al. 2024	*de novo*		field	6.12 × 10^− 7^	55	57/255	GLM
*Lalb_Chr06*	7,243,029	Schwertfirm et al. 2024	*de novo*		field	1.99 × 10^− 6^	65	47/255	GLM
*Lalb_Chr06*	7,349,762	Schwertfirm et al. 2024	*de novo*		field	1.99 × 10^− 6^	51	67/255	GLM
*Lalb_Chr08*	11,779,503	Schwertfirm et al. 2024	*de novo*		field	1.00 × 10^− 7^	45	254/255	GLM
*Lalb_Chr10*	15,808,902	Schwertfirm et al. 2024	*de novo*		field	4.88 × 10^− 8^	43	255/255	GLM

^a^ – “*de novo*” stands for markers identified in the present study, while “published” characterize markers from literature

After GWAS, the seven most significant SNPs with highest explained variance values ranging between 0.32 and 0.65 (Table 2) across all phenotypic data available (three phenological stages from field scorings) were selected for PACE assay development, irrespective of missing marker information. Two markers were overlapping with the ones presented in the original publication, while five additional ones were defined due to the additional models used and due to looser selection criteria (Supplementary Table S5).

Allelic states at the QTL/GWAS derived loci (Table 2) were analysed individually to determine significant association of the respective phenotypic values with analysis of variance (ANOVA) and post-hoc TukeyHS using the package stats version 3.6.2 (R Core Team, 2024) or with t-test to test the statistical difference (*p* < 0.05), depending on whether there are three or two allelic groups at the tested locus. Explained variance values were calculated independently for each marker locus as the proportion of the total sum of squares (SS_total_) attributable to the marker (SS_marker_), expressed as a percentage:

$$\:Explained\:Variance\:\left(\%\right)=\frac{{SS}_{marker}}{{SS}_{total}}\:\times\:100$$

For the estimation of overall performance of the set of markers individually associated to the phenotype (Table 2) phenotypic variance explained (PVE) values were calculated and compared. PVE values for all validation panels were calculated (adjusted R^2^ approach) using the package CJAMP version 0.1.1 (R Core Team, 2024), using a SNP only if it was polymorphic in the respective dataset.

### New markers from genomic prediction re-analysis of Alkemade et al. (2022) dataset

Genomic prediction was newly conducted on the Alkemade et al. (2022) dataset (raw data as provided in the original study) in R version 4.2.1 (R Core Team, 2022) using *rrBLUP* version 4.6.1 (Endelman 2011).

Missing genotypic information was imputed using the function A.mat with mean imputation method and a maximum missing marker information of 0.5. Genomic prediction was tested in a cross validation set-up of combinations of different (i) genotype numbers (ranging from 50 to 170), (ii) percent of training from 0.3 to 0.9 increasing by 0.1 increments; (iii) phenotypic values from controlled conditions presented in Alkemade et al. (2022), namely AUDPC and sAUDPC, running 20 iterations for each cross validation. The (AUDPC)^2^ value was found to be most descriptive based on skewness, thus was selected as the variable for marker definition, but marker effects and predicted phenotypic values were re-calculated with the sAUDPC score, which is more consistent with literature.

After running the GP with all genotypic data included, several rounds of marker thinning to achieve a relevant, yet minimized SNP-marker panel, were performed as follows: first, initially running the GP and omitting markers below the absolute cut-off value with minimal effect sizes for consideration (being minimum/maximum marker effect multiplied by 0.6). The GP was repeated using the thinned marker list and its performance was compared to a randomly sampled marker set of the same marker density to identify most relevant markers. Following, additional rounds of thinning and comparisons were applied by dropping markers one at a time and rerunning the GP to check how the prediction accuracy changed. If the change in correlation was neutral or had a negative impact on the prediction accuracy, that marker was omitted from the marker set due to irrelevance, which thinning step was repeated three more times. Subsequently, by running ANOVA tests, *p*-values (*p*) were estimated for variations among the two allelic groups and explained variance values were calculated for each marker. Markers with *p* < 0.005 and explained variance > 10% were designated as highly relevant, and the best six were used to generate a genome-wide haplotype. Using these haplotypes, *p*- and explained variance values were estimated once more with haplotypes with *p* < 0.005 and explained variance > 70% designated as relevant. These six markers, with the remaining markers from the last marker thinning step were used for subsequent assay design and genomic prediction, selecting 50 SNP markers in total.

This resulted in 50 highly informative markers. However, six of them contained too many polymorphic loci in the context sequence to design PACE assays, and two additional more failed at the PACE assay reaction step due to ambiguous amplification confirmed by a melt step. These eight SNPs represented marker effects ranging between − 0.0196 and 0.0202. Therefore, a final GP model, based on the technically working 42 markers, (Table 3, Supplementary Table S7) was used in our study and compared for performance in the training and validation sets.

**Table 3:** Distribution of 42 SNP markers used for genomic prediction across the 25 chromosomes of white lupin. Detailed information on the loci is presented in Supplementary Table S7

Chromosome	Marker number
*Lalb_Chr01*	4
*Lalb_Chr02*	5
*Lalb_Chr04*	4
*Lalb_Chr05*	1
*Lalb_Chr06*	3
*Lalb_Chr10*	4
*Lalb_Chr11*	2
*Lalb_Chr13*	1
*Lalb_Chr14*	3
*Lalb_Chr15*	2
*Lalb_Ch17*	4
*Lalb_Chr18*	4
*Lalb_Chr19*	1
*Lalb_Chr20*	1
*Lalb_Chr21*	1
*Lalb_Chr22*	1
*Lalb_Chr23*	1

### Genomic prediction model application and validation

42 loci (Table 3) were used for genomic prediction performance evaluation, in the three validation panels. The analysis, as for the model training, is based on a ridge regression, conducted in R version 4.4.2 (R Core Team, 2024) using rrBLUP version 4.6.3 (Endelman 2011). For validation using the original training dataset, missing allelic information was imputed using A.mat with the mean method with maximum missing marker information set to 0.1. The imputed allelic matrix was multiplied by the respective marker effect, and the values were adjusted by the beta, both predefined during model training using the training panel`s phenotypic values with the 42 successful SNP-marker genotyping data. The genomic prediction was performed with 20 k-fold cross-validations in the original training datasets. Due to sample size in the validation panels, 10 k-fold cross-validations were performed for the multi-parental panel (field phenotyping, *n* = 96), and 5 k-fold cross-validation for the diversity- (*n* = 48), multi-parental- (controlled conditions phenotyping, *n* = 22) and biparental (*n* = 50) panels. Mean and standard error of the correlation (Pearson correlation, r) between predicted and observed phenotypic values, root mean square deviation (RMSE, Sharma et al. 2022) and coefficient of determination (R^2^, Wright 1921) values were calculated across cross-validations.

In addition, improved genomic prediction models included retraining with the different panels using the same cross-validation approach as described above. The retrained datasets consisted of data from one validation panel, merged together with the data from the initial training panel for a total of four re-trained models (Table 4). For datasets originating from controlled conditions, adjusted phenotypic scores were recalculated for the merged retraining dataset as described above, while for those from the field, the original adjusted phenotypic values from the training panel were merged with the field phenotypic values of the respective dataset.

The allelic states of the 42 SNP markers were also used for a principal component analysis (PCA) to highlight intra- and interpopulation structure, created using *PCAtools* version 2.14.0 (Blighe and Lun 2023).

## Results

### Anthracnose resistance in diversity, multi-parental and biparental panels

No accession showed full resistance to anthracnose, neither in the climate chamber, nor in the field. Both diversity- and multi-parental panels showed clear differentiation between highly susceptible accessions and most resistant ones, with a continuous range of values for the accessions in between (Supplementary Table S1, Supplementary Table S3).

**Fig. 1:**
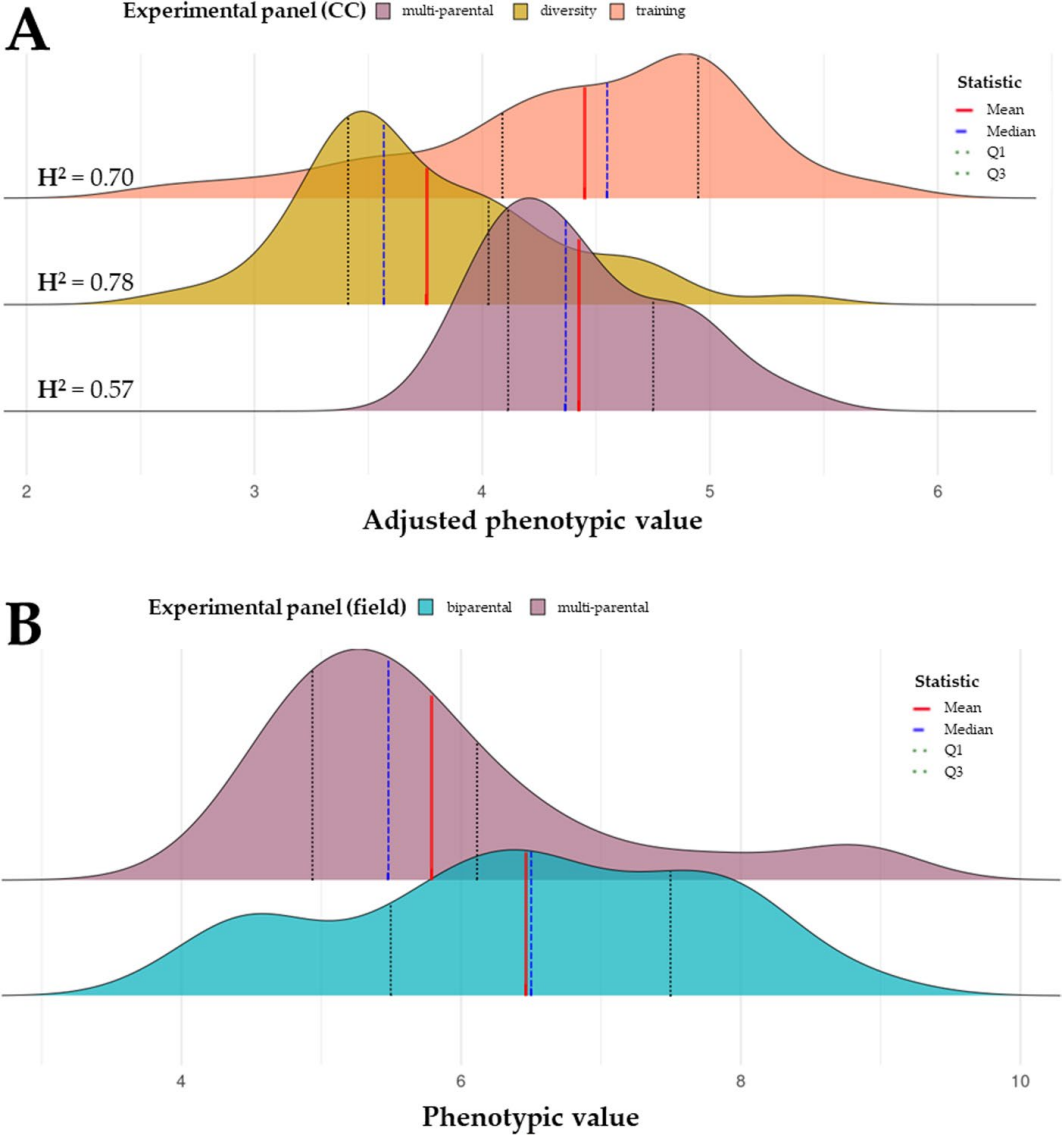
Ridge plots of the distribution of phenotypic data of the validation panels, marking mean (red line), median (blue dashed line), Q1 and Q3 (black dotted lines) values in each dataset. (**A**) Ridge plots of the adjusted sAUDPC values (6 biological replicates) originating from climate chamber trials (**B**) Ridge plots of the phenotypic values (single plant observations) originating from field observations, sAUDPC values for the multi-parental panel (reddish brown) and a single late-season disease score for the biparental pane (blue). The broad sense heritability (H^2^) value is marked in each sub-plot

The diversity panel (Fig. 1A, yellow) was curated to represent the span of resistance status of the global genepool, but focusing on enriching resistant accessions, which are scarce. The adjusted sAUDPC scores ranged from 2.65 (± 0.28) to 5.37 (± 0.28), with three breeding lines from the FiBL-gzpk breeding programme surpassing the most resistant variety on the market, `Frieda` (Supplementary Table S1). In the multi-parental panel`s phenotypic data from controlled conditions (Fig. 1A, reddish brown), the values ranged from 3.84 (± 0.30) to 5.29 (± 0.23) (Supplementary Table S3). The cultivar `Frieda` performed worse than most of the breeding lines (sAUDPC score = 4.67 ± 0.22), and `Amiga` performed the worst out of all included accessions. Conversely, in the field (Supplementary Table S2, Fig. 1B, reddish brown), the multi-parental panel ranged between 4.05 and 7.85 (sAUDPC scores evaluated for individual plants in this case), and `Frieda` performed the third best (sAUDPC score = 4.25). The F_3_ individual plants in the biparental panel (Supplementary Table S4, Fig. 1B, blue) showed great differentiation in anthracnose (single late-season disease score) values ranging from 4 to 9.

Within our multi-parental panel, comparing the field and controlled conditions observations, the phenotype of individuals in the field and the progeny of 22 of the exact same plants under controlled conditions did not correlate (*p* = 0.718). However, the correlation value between controlled conditions and field phenotyping (plot level) in a subset of 12 accessions from the training panel used for the genomic prediction where both scorings were available was 0.75 (*p* = 0.004).

Broad sense heritability values for the experiments from controlled conditions (where biological replication was available) were moderate to high, 0.70 for the training panel, 0.78 for the diversity- and 0.57 for the multi-parental panel (Fig. 1A).

### Testing marker-trait associations from literature in diversity- and breeding panels

**Fig. 2:**
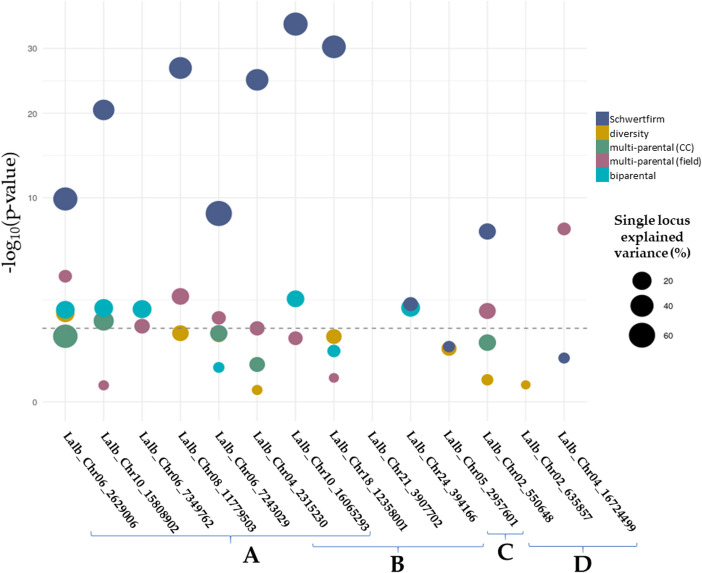
Scatter plot scaled to size of the proportion of variance explained of SNP-markers from literature and newly identified. Marker names on the x-axis refer to the individual SNPs and, on the y-axis their respective -log_10_(*p*-value) from analysis of variance of phenotypic value across the allelic states, with significance threshold of *p* = 0.05 (dotted line). Phenotypic variance explained (PVE) values were calculated for the validation panels based on all individual SNP markers. (**A**) 8 markers originating from the present study, using the raw data from Schwertfirm et al. (2024). (**B**) 5 high confidence SNP-markers originating from Schwertfirm et al. (2024) with successful PACE-assays out of the 7 targeted. (**C**) 1 marker originating from Alkemade et al. (2022). (**D**) 3 markers originating from Rychel-Bielska et al. (2020). Colours show which of the evaluation panels the dot refers to” (navy – original dataset presented in Schwertfirm et al. 2024; yellow – diversity panel; green – multi-parental panel using phenotypic data from the climate chamber; reddish brown – multi-parental panel using phenotypic data from the field; light blue – biparental panel), and the size refers to the explained variance value

Of the three markers reported by Rychel-Bielska et al. (2020) (Table 2), both Lalb_Chr02_550648 and Lalb_Chr04_16724499 were present in the raw SNP dataset reported by Schwertfirm et al. (2024); however, only Lalb_Chr02_550648 was significantly associated with anthracnose score in that dataset (*p* = 1 × 10⁻⁷). Lalb_Chr02_550648 also showed significant association with the phenotype in the multi-parental panel based on field observations (*p* = 0.01; Fig. 2D; Supplementary Table S7). The marker Lalb_Chr02_635857 was monomorphic in all datasets except the diversity-panel, where it showed no significant association with the phenotype (*p* = 0.85).

Allelic groups at Lalb_Chr05_2957601, a locus selected from the three reported by Alkemade et al. (2022), were not associated with differences in anthracnose severity in any of the three evaluated panels (Fig. 2C).

The performance of five high-confidence SNP markers identified by Schwertfirm et al. (2024) was further assessed across all validation panels and compared with their performance in the original dataset. Across all validation panels, we observed a consistent reduction in explained variance and strength of association relative to the original results (Fig. 2B). Regarding polymorphism, three markers were polymorphic in the diversity-panel, two in the multi-parental panel under climate chamber conditions, three in the multi-parental panel based on field data, and four in the biparental panel. In terms of association with anthracnose severity, none of the markers showed significant associations in either the diversity-panel or the multi-parental panels. In contrast, two of the four polymorphic SNPs in the biparental panel (Lalb_Chr24_394166 and Lalb_Chr10_16065293) were significantly associated with disease score at a threshold of *p* = 0.05 (Fig. 2B).

Two loci overlapped between the original GWAS reported by Schwertfirm et al. (2024) and the GWAS conducted in the present study on the same dataset (Lalb_Chr04_2315230, Lalb_Chr10_16065293). Of these, none showed significant associations with anthracnose severity in the validation populations, except Lalb_Chr10_16065293, which was significantly associated in the biparental panel (*p* = 0.005; Fig. 2A–B overlap; Supplementary Table S7).

Using all genotypic data of individually assessed loci together to calculate the total phenotypic variance explained (Fig. 2), the model explains 21% and 24% of the observed phenotypic variance in the field panels. For the diversity panel, although the trait shows high broad-sense heritability (H^2^ = 0.78, Fig. 1A), the fitted single marker-based model using the individually assessed markers together, captures only a modest proportion of total phenotypic variance (PVE = 15%). For the multi-parental panel, moderate-to-high heritability (H^2^ = 0.57, Fig. 1A) comes with a higher PVE (PVE = 26%).

### Testing newly identified marker-trait associations in diversity- and breeding panels

Apart from the two aforementioned loci overlapping with the original publication, this study identified five additional SNP-markers (Fig. 2A), originating from the re-analysis of the dataset published in Schwertfirm et al. (2024). This re-analysis consisted of a complementary GWAS to the original dataset presented.

**Fig. 3:**
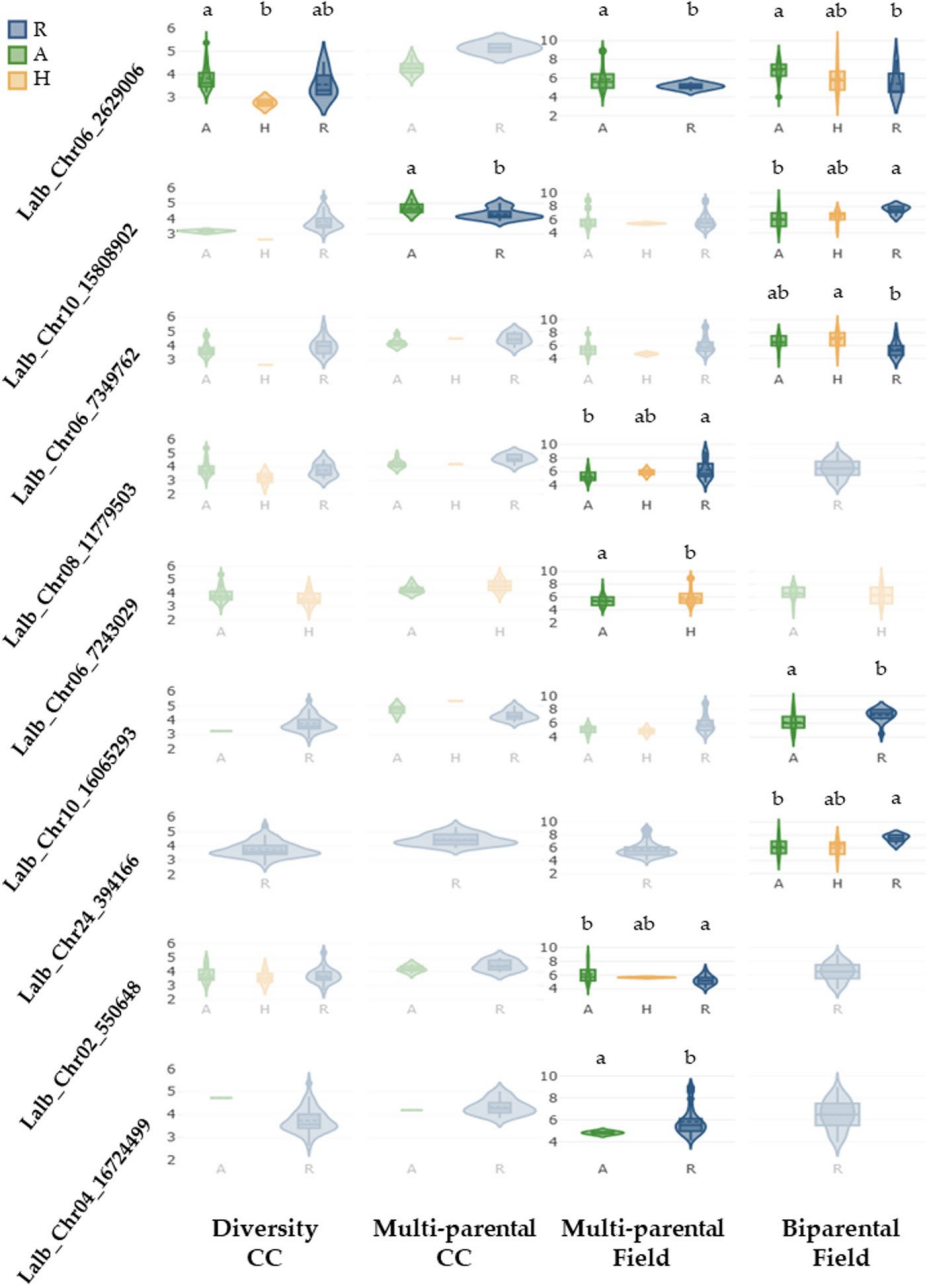
Violin plots of different allelic states and their associated phenotypic values (sAUDPC on the y-axis) with statistical grouping in case of significant association in at least one validation panel. The colour blue refers to individuals carrying the reference allele (R, allelic state associated to the reference genome of the cultivar `Amiga`), green to the individuals carrying the alternative allele (A), and yellow to heterozygous individuals (H). Blurred windows show no significant variations between the alleles in regard to the anthracnose level. Letters above the clear window allele comparisons indicate significance groups (*p* < 0.05)

In the validation, no single marker was consistently associated to the anthracnose trait within all the investigated panels. However, Lalb_Chr06_2629006 was significantly associated to the disease phenotype in the diversity-, multi-parental- (field phenotyping) and the biparental panel with single locus explained variance values of 0.18, 0.03 and 0.18 respectively, with clear differences between different allelic groups in the latter two panels (Fig. 3). One additional SNP-marker Lalb_Chr10_15808902 was significant in the multi-parental (climate chamber phenotyping) and the biparental panels, but association of the allelic state was inconsistent across the two cases. Three additional SNP-markers from the five newly identified loci had a significant association to the anthracnose phenotype in at least one validation panel. Two located on chromosome *Lalb_Chr06* (Lalb_Chr06_7349762, Lalb_Chr06_7243029), and one on chromosome *Lalb_Chr08* (Lalb_Chr08_11779503), with significant associations with field observations (multi-parental and biparental panel, Fig. 3).

### Testing accuracy of genomic prediction model based on a minimal number of markers

As for diversity, compared to the 42 markers selected for genomic prediction in the training panel (original dataset from Alkemade et al. 2022), all are polymorphic in the diversity-panel, while 39 and only 16 are polymorphic in the multi-parental and in biparental panel, respectively.

**Fig. 4:**
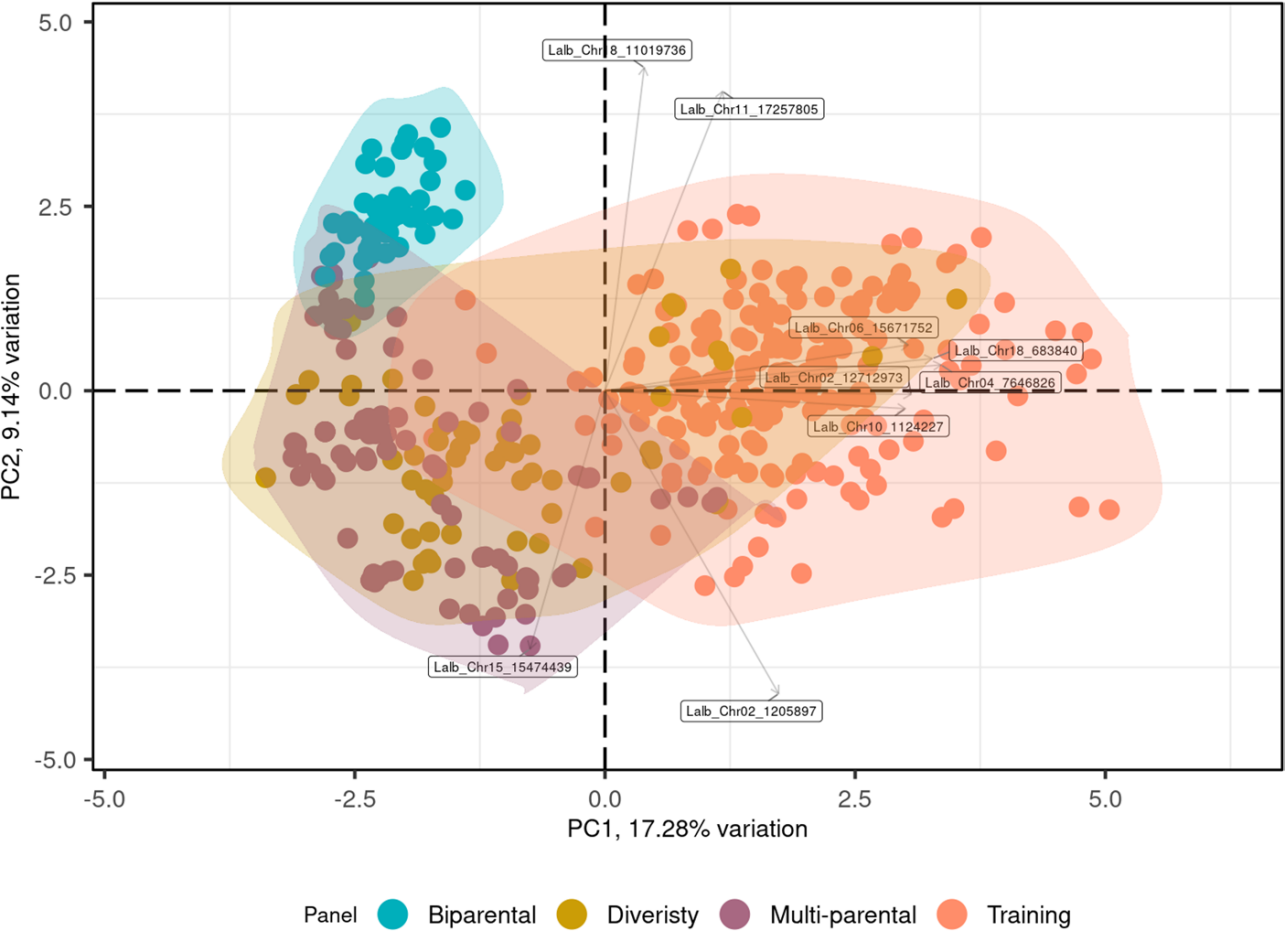
First two principal components (PC) of PCA representing all datasets used in the study, colours, shapes and encircling indicate different panels. All SNP-markers (*n* = 42) included in the analysis

The first two components of the PCA (Fig. 4), explaining 17.28% and 9.14% of variation using all 42 markers (PC03-37 ranging from 6.1 to 0.3%), reveal that the training panel is closer related to the diversity-panel compared than to the multi-parental panel. The individuals of the multi-parental panel, clustering together with those of the training panel, represent three independent breeding lines carrying resistance sources from Chilean breeding programme and Ethiopian genetic resources, which resistance sources were present in the training panel as well (Fig. 4). Noticeably individuals of the biparental panel were clustering most distantly from the training panel.

**Fig. 5:**
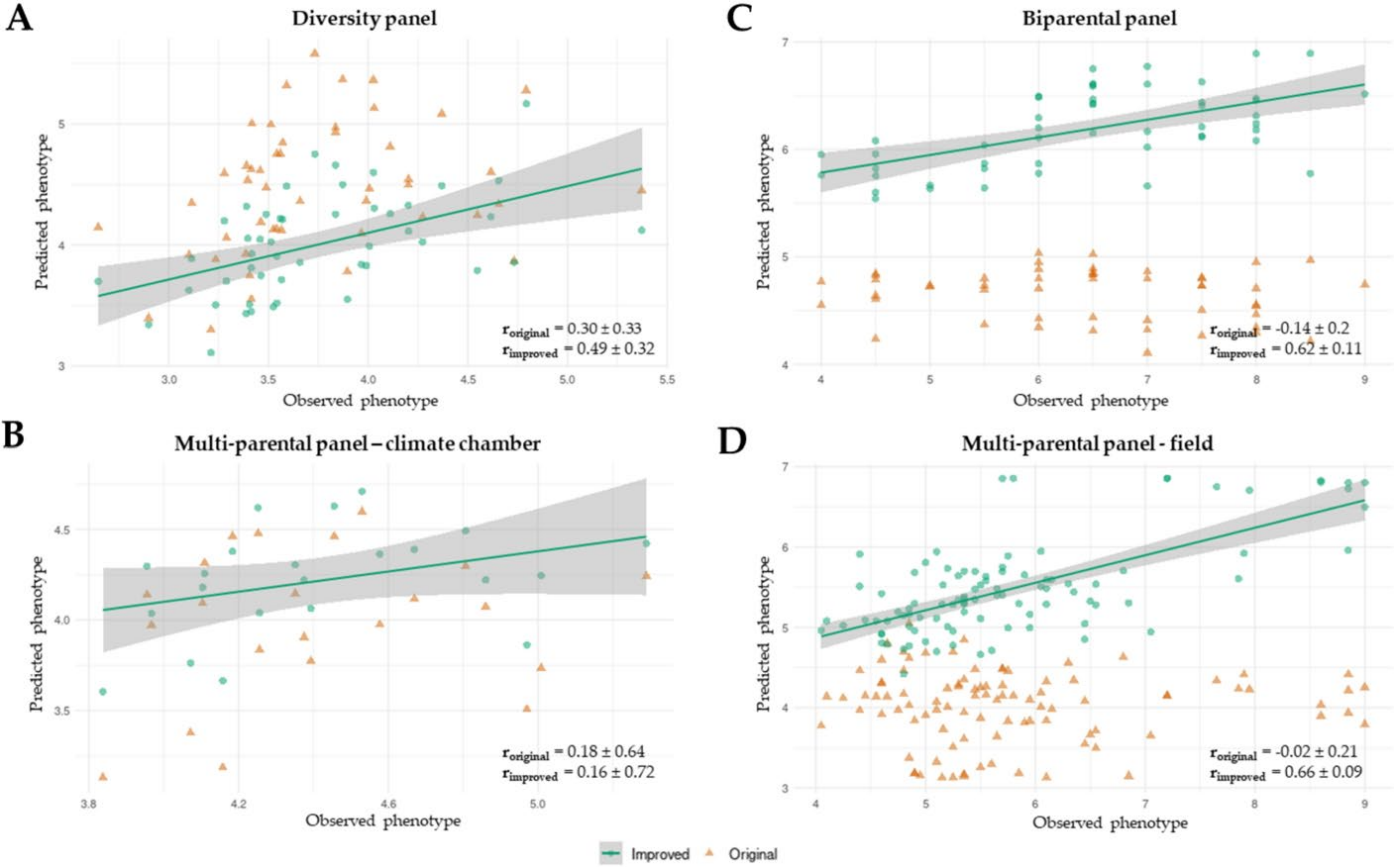
Scatter plots of predicted and observed phenotypic values, presenting original (r_original_) and improved (r_improved_) prediction accuracy. (**A**) Scatter plot of predicted and observed phenotypic values in the diversity panel (*n* = 48), using 42 polymorphic SNP-markers. (**B**) Scatter plot of predicted and observed phenotypic values in the multi-parental panel using the phenotypic value from controlled conditions (*n* = 22), using 39 polymorphic SNP-markers. (**C**) Scatter plot of predicted and observed phenotypic values in the biparental panel using the phenotypic value from the field (*n* = 50), using 16 polymorphic SNP-markers. (**D**) Scatter plot of predicted and observed phenotypic values in the multi-parental panel using the phenotypic value from the field (*n* = 96), using 39 polymorphic SNP-markers

The genomic prediction based on the 42 markers set had a prediction accuracy (*r*) of 0.84 ± 0.13 across 20 cross-validations in the training panel. The coefficient of determination (R^2^ = 0.58 ± 0.34) also signalled that the model accurately predicts the phenotypes, although with high levels of variation amongst cross-validations (Fig. 5A; Table 4). In the diversity-panel (42 markers), the correlation between predicted and observed phenotypic values across 5-fold cross-validations had a prediction accuracy of *r* = 0.30, with high standard error across cross-validations (SE = 0.33). Although the RMSE value (0.95 ± 0.07) was within acceptable range, the coefficient of determination R^2^ = −2.89 ± 1.89 highlights the failure of the initial model (Fig. 5B; Table 4). There is no significant correlation between predicted and observed values for the multi-parental panel (39 polymorphic loci, Fig. 5C-D) and the biparental panel (16 polymorphic loci, Fig. 5E; Table 4).

**Table 4:**
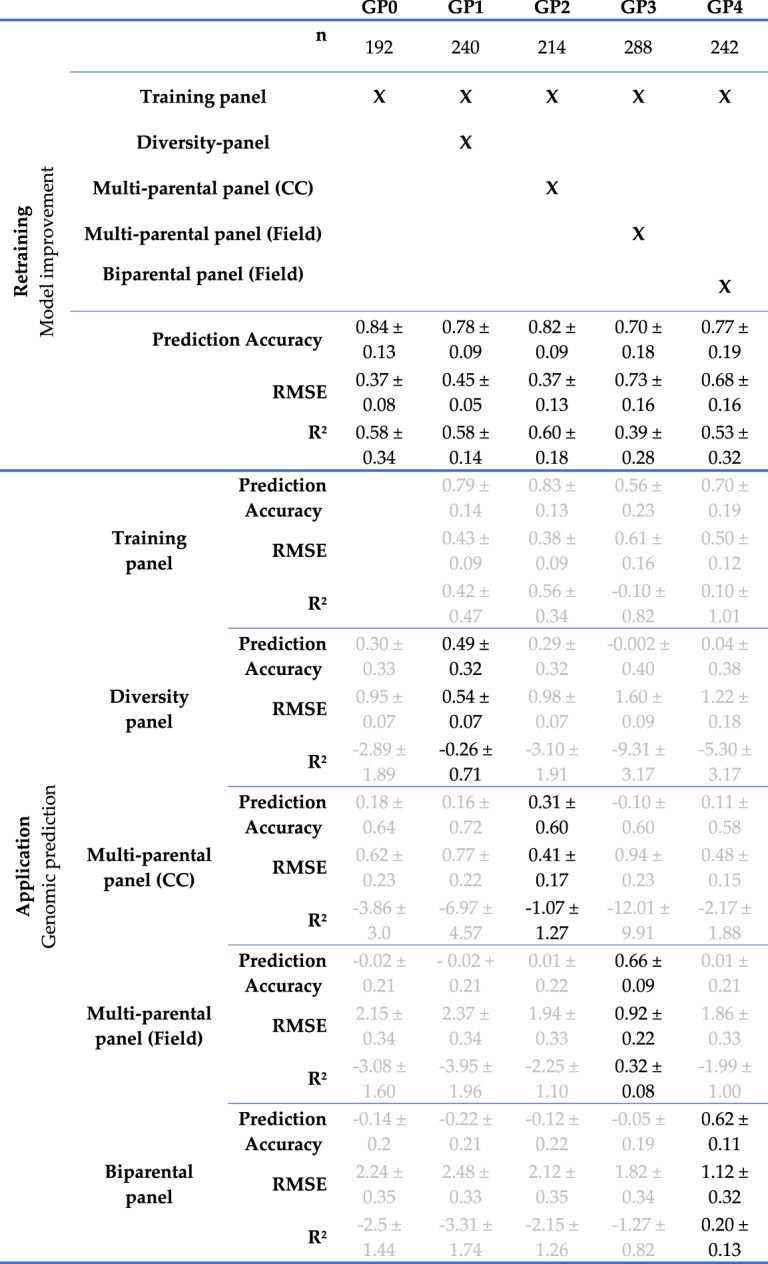
Prediction accuracy (r), RMSE and R^2^ values and respective standard error of retrained GP models (GP1-GP4) and of the original model (GP0), as well as the aforementioned metrics for genomic prediction in all panels included in the study. Each column represents a different combination of validation panels used for model training together with the training panel. 20-fold cross validations were performed for all training datasets, 10-fold for validation in the multi-parental panel (field observations), and 5-fold for the remaining models. Results of the respective dataset used for re-training in black, other combinations in dark grey

### Retraining genomic prediction model enhances prediction accuracy

As the initial estimated genomic prediction model was unsuitable to make predictions beyond the genomic diversity boundaries it was trained on, we aimed to improve the genomic prediction model. Therefore, we made use of the additional source of diversity found in the diversity-, multi-parental and biparental panels, keeping the same 42 markers (Table 4). For GP1 and GP2 adjusted sAUDPC values were used, while for GP3 and GP4, phenotypic values from the climate chamber and the sAUDPC values from the field observations were merged before the model training.

Genomic prediction performance was evaluated by comparing the original model trained on the training panel alone (Fig. 5; Table 4, GP0) with models retrained using merged datasets (Fig. 5; Table 4, GP1–GP4). Based on 20-fold cross-validation within the respective training datasets, the highest prediction accuracy was obtained using the training panel only (*r* = 0.84 ± 0.13). All retrained models showed stable predictive performance, albeit with slightly reduced accuracy (Fig. 5; Table 4, GP1–GP4).

When applied to the original training dataset, retrained models exhibited comparable performance, with similarly low mean RMSE values and comparable coefficients of determination. Models retrained with datasets derived from controlled phenotyping conditions (Fig. 5A-B; Table 4, GP1–GP2) showed reduced standard errors across cross-validations, indicating increased prediction stability. In contrast, models retrained using datasets that included field-phenotyped panels (Fig. 5C-D; Table 4, GP3–GP4) displayed increased mean RMSE values and greater variability, consistent with the higher phenotypic variance observed under field conditions (Fig. 1B). The coefficient of determination remained high when the training panel was merged with the biparental panel (Table 4, GP4), whereas a reduction in R² was observed when merged with the multi-parental panel field phenotypes (Table 4, GP3).

Retraining using field-derived phenotypic datasets resulted in effective prediction within their respective validation panels. For the multi-parental panel, prediction accuracy reached r_improved_ = 0.66 ± 0.09 with a coefficient of determination of R² = 0.32 ± 0.08 (Fig. 5D; Table 4, GP3). The corresponding mean RMSE (0.92 ± 0.22) reflected the broader phenotypic range present in field observations. Similarly, the model retrained with the biparental panel exhibited a prediction accuracy of *r* = 0.62 ± 0.10 and R² = 0.20 ± 0.13, accompanied by higher RMSE values (1.12 ± 0.32), consistent with increased phenotypic dispersion (Fig. 5C; Table 4, GP4).

In contrast, retraining did not consistently improve predictive performance for validation panels with phenotypes originating from controlled conditions. The model retrained with the diversity panel (Fig. 5A; Table 4, GP2) showed substantial variability across cross-validations (*r* = 0.49 ± 0.32) and limited predictive ability. Although mean RMSE values remained stable (0.54 ± 0.07), coefficients of determination were highly variable and frequently negative (R² = −0.26 ± 0.71), indicating poor model transferability in this scenario.

## Discussion

Our analysis of the performance of anthracnose resistance markers in the context of a small- scale white lupin breeding programme, aimed to close the gap between advanced molecular marker research and practical implementation of the results in plant breeding, while highlighting the challenges involved. For easy application in molecular marker-assisted selection, we worked with the cost-effective PACE genotyping, suitable also for small sized breeding programmes such as the organic one in Switzerland by FiBL and gzpk (a non-profit plant breeding association with more than 30 years of experience in organic plant breeding).

Our validation panels consisted of two breeding panels from the aforementioned breeding programme, and a diversity panel. The diversity-panel was curated to represent the span of the phenotypic variability of the trait, maximising the presence of phenotypically resistant accessions. This was meant to complement and validate the findings of the GWAS studies presented on the two panels (LUW and training) presented by Alkemade et al. (2022) and Schwertfirm et al. (2024). Comparing the broad sense heritability (H^2^), we observed (Fig. 1A) high values for the training and diversity-panels (0.70 and 0.78, respectively). However, in the diversity-panel, only a limited fraction of the total phenotypic variance (PVE = 15%) is captured by the predictors included in the model, highlighting the limitation of genetic architecture of training vs. validation panels. This limitation is also observed in the multi-parental panel using the climate chamber values (H^2^ = 0.57, PVE = 26%).

Individually assessing SNP loci described in previous publications, no single marker from literature was consistently successful in discriminating the anthracnose disease level across all panels used for validation. The most significant locus from the study by Schwertfirm et al. (2024), Lalb_Chr10_16065293 (-log(*p*) = 14.91), was significant in the biparental panel, where one of the parents is the cultivar `Frieda`. This is particularly interesting since 41 out of 73 breeding lines from the panel of the original study clustered together with the cultivar `Frieda`, leading to the assumption that the resistance source present in `Frieda` is overrepresented in this panel, thus the marker-trait association presented there would also contain the same source of resistance. This specific SNP on chromosome *Lalb_Chr10* is also in the QTL region that the latest white lupin linkage map presents for anthracnose resistance (Książkiewicz et al. 2017), a finding consistent with ca. ten years of research. However, it is not located in the coding sequence of any gene, rather within a mRNA cluster (cluster_56277), which are known epigenetic regulatory molecules potentially associated to plant defence (Yang et al. 2021; Luo et al. 2024; Mierziak and Wojtasik 2024). We found no paralogs or orthologs through nBLASTs (Altschul et al. 1990; Boratyn et al. 2012; Camacho et al. 2009; Morgulis et al. 2008; Zhang et al. 2000), thus no further information could be concluded regarding its function or involvement in defence against fungal pathogens.

As a second step, this study aimed to expand the utility of already existing datasets with the re-analysis for further marker discovery through GWAS in the dataset Schwertfirm et al. 2024 and through GP in the dataset Alkemade et al. (2022).

Schwertfirm et al. (2024) present a GWAS based on multi-locus models (MLMM, FarmCPU and BLINK) applied to the pre-filtered marker set for missing data and minimum allele frequency. The re-analysis in our study aimed to focus on SNPs which were lost during the filtering steps done in the original publication. GLM enabled the inclusion of these markers, preserving potentially informative genetic variation (Kaler et al. 2020). The approach is considered robust as the performance of all newly identified markers was assessed in all environments and populations included for adequate validation.

All five additional SNP-markers from the repeated GWAS are significant in at least one validation panel, with one marker (Lalb_Chr06_2629006) being significant in three out of the four different validation panels highlighting the specificity based on the genetic origin of the resistance.

To exploit a different source of markers and selection approach, we performed genomic prediction in the data set by Alkemade et al. (2022). We developed a model based on a limited number of 42 markers as blue-print for low-cost application of genomic selection in small scale breeding programmes for hard to phenotype, polygenic traits. There was no possibility to cross-validate findings from the two derived SNP datasets (Alkemade et al. 2022; Schwertfirm et al. 2024) due to the poor overlap between raw SNPs, as only 14 loci within the 42 marker panel could be found in the dataset of Schwertfirm et al. (2024).

Compared with the high prediction accuracy of the original genomic prediction (GP) model in the training panel (r_original_ = 0.84 ± 0.13), predictive performance dropped substantially in all independent validation panels. In these panels, correlations between predicted and observed anthracnose scores were close to zero, and in several cases the coefficient of determination (R²) was negative, indicating poor and unstable prediction. A major limitation affecting model transferability is the reduced allelic diversity in the validation panels relative to the training population. Of the 42 SNP markers included in the original model, only 39 were polymorphic in the multi-parental panel and only 16 in the biparental panel. This reduction reflects the narrower genetic base of the white lupin breeding panels and limits the model’s ability to capture the full range of marker effects identified during training. Recalculating marker effects using only the polymorphic markers (39 or 16 SNPs, respectively) did not improve predictive performance, suggesting that the loss of informative loci—and not simply incorrect estimation of marker effects—contributes to the observed decline in accuracy. Even in the diversity-panel, which was genetically most similar to the training panel (Fig. 4) and in which all 42 markers were polymorphic, prediction accuracy remained highly variable (r_original_ = 0.30 ± 0.33) with coefficient of determination values consistently negative (R² = −2.89 ± 1.89). This instability may be connected to the enrichment of resistance sources, determined by phenotyping results rather than genotyping data in the diversity-panel compared to the training panel. Differences in sample size, trait distribution, and phenotypic variance between the training and validation datasets likely further reduced model performance. In addition, mismatches between phenotyping environments may have contributed to the poor transferability of the GP model. Validation panels included data from controlled environments and field conditions that differed from those used for model training (controlled conditions only), potentially altering the expression of quantitative resistance and the relevance of specific marker effects. Marker–environment interactions may therefore play an important role in limiting model stability. Overall, the consistently low predictive ability observed across validation panels (Table 4, GP0) highlights the combined impact of limited panel diversity, environmental heterogeneity, and marker–environment interactions on GP model robustness.

Most GP models use a much higher marker density, although the increase in available marker information does not always correlate with increased prediction accuracy (Fu et al. 2017; Zhao et al. 2025). In a recent study about genomic prediction in white lupin (Annicchiarico et al. 2025), the authors present a genomic selection model for key agronomic traits of seed weight, protein- and oil content as well as total quinolizidine alkaloid content focusing on intra- and inter-population, as well as intra- and inter-environment scenarios. Despite of using over 30,000 SNPs for prediction in all scenarios, low prediction accuracy scores were observed in inter-population and inter-environment scenarios when comparing breeding lines to landraces, consistent with our findings.

From another pathosystem, Juliana et al. (2017) report analyses for leaf-, stem- and stripe rust resistance in wheat, using 5,102 and 8,066 marker loci for the prediction, respectively. Even with the magnitudes higher marker density than presented in this study, the prediction accuracy was 0.39 when working with one wheat dataset. A different study on wheat focused on *Fusarium* head blight for which the resistance (similarly to anthracnose resistance in white lupin) is polygenically controlled. Using 4,205 SNPs for the genomic prediction, the authors report prediction accuracies ranging between 0.4 and 0.6 for disease severity and -incidence using *rrBLUP* (Zhang et al. 2021). In the present study, four sequential marker thinning steps were included to reach 42 highly informative markers, and the prediction accuracy dropped substantially from the training to the diversity panel. In a study made on Norway spruce, the authors test the limitations of genomic prediction models by decreasing marker densities looking at different phenological characteristics. For bud burst, they report a prediction accuracy of 0.63 using 4,000 SNPs, which number decreased to 0.36 when using the allelic information of only 25 SNPs (Chen et al. 2023), which phenomenon was also reported in Cerioli et al. (2022) when using genomic prediction with systematically decreasing marker densities for agronomic traits in rice. Although, using a larger marker density does not per se correlate to more accurate predictions. For example, in a genomic prediction study for soybean mosaic virus resistance in soybean, researchers identified more accurate prediction accuracy using the top 100 loci, compared to the more than 8,000 available SNPs. The inclusion of all genotypic data available could create unwanted background noise (Zhao et al. 2025), potentially improved by continuous marker thinning steps. In particular, the approach used in this study (ridge regression), rely on effect shrinkage toward 0. Shrinking marker effects for predicting the phenotype of interest is the core strategy to limit false predictions. The more markers unrelated to the phenotype of interest are implemented in such models, the more shrinkage of marker effects needs to be performed to limit errors – resulting in suboptimal models. Other approaches, likes BayesB or lasso approaches might allow to reduce marker effects to 0 instead of a very small value – nevertheless, these models in general do not lead to drastically different prediction accuracies (Meuwissen et al. 2009; Puthiyedth et al. 2025). Therefore, “feature”, or marker selection, remains a robust approach to improve genomic prediction models. Although the minimal marker set showed to be successful in the training panel, it lacked predictive power in the validation panels, regardless of genetic background or panel size. This is not unexpected, especially considering that genomic prediction studies most often use subset-, interset- or progeny validation (Alemu et al. 2024; Crossa et al. 2025; Sallam et al. 2015) and higher marker density for complex traits, particularly in species with low linkage disequilibrium such as white lupin (Alemu et al. 2024). Increasing the marker quantity from 42 to 100 might have resulted in improved predictions but it was beyond the scope of this study. Another approach to improve the usefulness of GP is to extend the genetic diversity.

Genomic prediction models were retrained with the addition of a specific validation panel to the data from the training panel (Table 4, GP1-4), to improve the genomic prediction both from the facets of relatedness and sample size. Although still unable to accurately predict amongst validation panels, the retraining accentuates the possibility to still apply genomic prediction within unrelated material, with the inclusion of the panel of accessions to be validated into the genomic prediction model. The biggest limitation in our study was size of the specific validation panel. The least successful retrained model when applied to its corresponding validation panels was with the multi-parental panel, using the phenotypic values from controlled conditions (*n* = 22), with the highest deviation in across the cross validations and minimal success even in the best scenario (Fig. 5B; Table 4, GP2). Moreover, narrower genetic diversity within both breeding populations and the diversity panel highlights the limitations. When retraining the model with the diversity panel (Fig. 5A; Table 4, GP1) included in the model training, the deviation remains high in the prediction accuracy (*r* = 0.49 ± 0.32), and the coefficient of determination (R^2^ = −0.26 ± 0.71), highlighting great differences between cross-validations. The barrier that the retraining overcame is applying genomic prediction models trained with phenotypic values originating from controlled conditions for validation in panels where observations were made on the field. The combination of training panel and the multi-parental panel (field observations) showed the greatest improvement (Table 4, GP3), with reliable prediction accuracy (*r* = 0.66 ± 0.09) and coefficient of determination (R^2^ = 0.32 ± 0.08) across cross-validations, where the moderately high RMSE values (0.92 ± 0.22) could be explained by greater differentiation in phenotypic values observed on the field. This successful application, to a lesser extent, is also highlighted by the results of the model retraining with the biparental panel (Table 4, GP4). This scenario accentuates successful application in breeding panels with low genetic diversity, such as an F_3_ panel originating from a biparental crossing, regardless of the differences in phenotyping approaches (sAUDPC vs. single disease score). Thus, to address the inconsistencies between observations made on the field and in controlled conditions, the best suggested approach would be using the combination of marker loci presented in Schwertfirm et al. (2024), which study worked with phenotypes coming from the field, and those coming from Alkemade et al. (2022), which included phenotypic evaluation under controlled conditions, and taking into account marker-environment interactions, as suggested by Skøt et al. (2024). With the combined approach using a selection of SNPs, the marker set would address environmental context-dependency intrinsically. Subsequently, using these loci for continuous selection would enable a more reliable genetic gain throughout the years.

## Conclusion

With an in-depth analysis of previously published and newly detected loci associated to anthracnose resistance in white lupin, we provide a thorough investigation on the efficiency of currently available markers for marker-assisted selection. We confirmed SNP-markers associated to the previously reported QTL on chromosome *Lalb_10*, tagging the source of resistance associated to the cultivar ‘Frieda’.

We also constructed a genomic prediction model based on a minimal number of 42 PACE markers for use in small-sized breeding programmes, partially positively validated in a diversity-panel, while highlighting the limitations in application when working with a narrower and unrelated genepool. The effect of training and validation set diversity, phenotyping environment, and marker–environment interactions on model stability are discussed against most recent GP literature. Our results show that a retraining of genomic prediction models leads to improved genomic prediction accuracy for anthracnose resistance, despite relatedness and different phenotyping platforms of validation panels, compared to the initial training dataset.

## Supplementary Information

Below is the link to the electronic supplementary material.

Supplementary File 1 (XLSX 155 KB)

## Data Availability

The authors used publicly available datasets available from supplementary material from Alkemade et al. (2022) and Schwertfirm et al. (2024). All data generated or analysed during this study are included in this published article and its supplementary information files.

## Abbreviations

CAPS: Cleaved Amplified Polymorphic Sequence
FiBL: Forschungsinstitut für Biologischen Landbau (Research Institute of Organic Agriculture)
GP: Genomic Prediction
GWAS: Genome Wide Association Study
gzpk: Getreidezüchtung Peter Kunz (Cereal Breeding Peter Kunz)
LfL: Bavarian State Research Center for Agriculture
LMM: Linear Mixed Model
MAS: Marker Assisted Selection
PACE: PCR Allelic Competitive Extension
PCR: Polymerase Chain Reaction
PVE: Phenotypic Variance Explained
RIL: Recombinant Inbred Line
rrBLUP: Ridge Regression Best Linear Unbiased Predictor
sAUDPC: Standard Area Under Disease Progress Curve
SNP: Single Nucleotide Polymorphism

## References

[CR1] Adhikari KN, Buirchell BJ, Thomas GJ, Sweetingham MW, Yang H (2009) Identification of anthracnose resistance in Lupinus albus L. and its transfer from landraces to modern cultivars. Crop Pasture Sci 60(5):472. 10.1071/CP08092

[CR2] Adhikari KN, Thomas G, Diepeveen D, Trethowan R (2013) Overcoming the barriers of combining early flowering and anthracnose resistance in white lupin (Lupinus albus L.) for the Northern Agricultural Region of Western Australia. Crop Pasture Sci 64(9):914. 10.1071/CP13249

[CR3] Alemu A, Åstrand J, Montesinos-López OA, Isidro y Sánchez J, Fernández-Gónzalez J, Tadesse W, Vetukuri RR, Carlsson AS, Ceplitis A, Crossa J, Ortiz R, Chawade A (2024) Genomic selection in plant breeding: Key factors shaping two decades of progress. Mol Plant 17(4):552–578. 10.1016/j.molp.2024.03.007 38475993 10.1016/j.molp.2024.03.007

[CR4] Alkemade JA, Messmer MM, Arncken C, Leska A, Annicchiarico P, Nazzicari N, Książkiewicz M, Voegele RT, Finckh MR, Hohmann P (2021a) A High-Throughput Phenotyping Tool to Identify Field-Relevant Anthracnose Resistance in White Lupin. Plant Dis 105(6):1719–1727. 10.1094/PDIS-07-20-1531-RE 33337235 10.1094/PDIS-07-20-1531-RE

[CR5] Alkemade JA, Messmer MM, Voegele RT, Finckh MR, Hohmann P (2021b) Genetic diversity of Colletotrichum lupini and its virulence on white and Andean lupin. Sci Rep 11(1):13547. 10.1038/s41598-021-92953-y 34188142 10.1038/s41598-021-92953-y PMC8242092

[CR6] Alkemade JA, Nazzicari N, Messmer MM, Annicchiarico P, Ferrari B, Voegele RT, Finckh MR, Arncken C, Hohmann P (2022) Genome-wide association study reveals white lupin candidate gene involved in anthracnose resistance. Theor Appl Genet 135(3):1011–1024. 10.1007/s00122-021-04014-7 34988630 10.1007/s00122-021-04014-7 PMC8942938

[CR7] Alkemade JA, Baroncelli R, Messmer MM, Hohmann P (2023) Attack of the clones: Population genetics reveals clonality of Colletotrichum lupini, the causal agent of lupin anthracnose. Mol Plant Pathol 24(6):616–627. 10.1111/mpp.13332 37078402 10.1111/mpp.13332 PMC10189766

[CR8] Alkemade JA, Patyi A, Arncken C, Lazzaro M (2025) Lupins: a remarkable protein crop battling anthracnose for a greener future. Plant Health Cases 2025:phcs20250008. 10.1079/planthealthcases.2025.0008

[CR9] Altschul SF, Gish W, Miller W, Myers EW, Lipman DJ (1990) Basic local alignment search tool. J Mol Biol 215(3):403–410. 10.1016/S0022-2836(05)80360-2 2231712 10.1016/S0022-2836(05)80360-2

[CR10] Altschul SF, Madden TL, Schäffer AA, Zhang J, Zhang Z, Miller W, Lipman DJ (1997) Gapped BLAST and PSI-BLAST: a new generation of protein database search programs. Nucleic Acids Res 25(17):3389–3402. 10.1093/nar/25.17.3389 9254694 10.1093/nar/25.17.3389 PMC146917

[CR11] Annicchiarico P, Osorio C, Nazzicari N, Ferrari B, Barzaghi S, Biazzi E, Tava A, Pecetti L, Notario T, Romani M, Crosta M (2025) Genetic variation and genome-enabled selection of white lupin for key seed quality traits. BMC Genomics 26(1):922. 10.1186/s12864-025-12048-0 41094367 10.1186/s12864-025-12048-0 PMC12522229

[CR12] Blighe K, Lun A (2023) PCAtools: PCAtools: everything principal components analysis. Doi:10.18129/B9.bioc.PCAtools (Version 2.14.0) [Computer software]. https://bioconductor.org/packages/PCAtools

[CR13] Boratyn GM, Schäffer AA, Agarwala R, Altschul SF, Lipman DJ, Madden TL (2012) Domain enhanced lookup time accelerated BLAST. Biol Direct 7:12. 10.1186/1745-6150-7-12 22510480 10.1186/1745-6150-7-12 PMC3438057

[CR14] Camacho C, Coulouris G, Avagyan V, Ma N, Papadopoulos J, Bealer K, Madden TL (2009) BLAST+: architecture and applications. BMC Bioinformatics 10:421. 10.1186/1471-2105-10-421 20003500 10.1186/1471-2105-10-421 PMC2803857

[CR15] Cerioli T, Hernandez CO, Angira B, McCouch SR, Robbins KR, Famoso AN (2022) Development and validation of an optimized marker set for genomic selection in southern U.S. rice breeding programs. Plant Genome 15(3):e20219. 10.1002/tpg2.20219 35611838 10.1002/tpg2.20219 PMC12806970

[CR16] Chen Z-Q, Klingberg A, Hallingbäck HR, Wu HX (2023) Preselection of QTL markers enhances accuracy of genomic selection in Norway spruce. BMC Genomics 24(1):147. 10.1186/s12864-023-09250-3 36973641 10.1186/s12864-023-09250-3 PMC10041705

[CR17] CPVO (2026) *CPVO Variety Finder.* https://online.plantvarieties.eu/

[CR18] Crossa J, Martini JWR, Vitale P, Pérez-Rodríguez P, Costa-Neto G, Fritsche-Neto R, Runcie D, Cuevas J, Toledo F, Li H, De Vita P, Gerard G, Dreisigacker S, Crespo-Herrera L, Saint Pierre C, Bentley A, Lillemo M, Ortiz R, Montesinos-López OA, Montesinos-López A (2025) Expanding genomic prediction in plant breeding: harnessing big data, machine learning, and advanced software. Trends Plant Sci 30(7):756–774. 10.1016/j.tplants.2024.12.009 39890501 10.1016/j.tplants.2024.12.009

[CR19] de Mendiburu F (2023) agricolae: statistical procedures for agricultural research (Version 1.3-7) [Computer software]. https://cran.r-project.org/web/packages/agricolae/index.html

[CR20] Endelman JB (2011) Ridge Regression and Other Kernels for Genomic Selection with R Package rrBLUP. Plant Genome. 10.3835/plantgenome2011.08.0024

[CR21] Fu Y-B, Yang M-H, Zeng F, Biligetu B (2017) Searching for an accurate marker-based prediction of an individual quantitative trait in molecular plant breeding. Front Plant Sci. 10.3389/fpls.2017.01182 28729875 10.3389/fpls.2017.01182 PMC5498511

[CR22] Gabur I, Chawla HS, Liu X, Kumar V, Faure S, von Tiedemann A, Jestin C, Dryzska E, Volkmann S, Breuer F, Delourme R, Snowdon R, Obermeier C (2018) Finding invisible quantitative trait loci with missing data. Plant Biotechnol J 16(12):2102–2112. 10.1111/pbi.12942 29729219 10.1111/pbi.12942 PMC6230954

[CR23] Hufnagel B, Marques A, Soriano A, Marquès L, Divol F, Doumas P, Sallet E, Mancinotti D, Carrere S, Marande W, Arribat S, Keller J, Huneau C, Blein T, Aimé D, Laguerre M, Taylor J, Schubert V, Nelson M, Péret B (2020) High-quality genome sequence of white lupin provides insight into soil exploration and seed quality. Nat Commun 11(1):492. 10.1038/s41467-019-14197-9 31980615 10.1038/s41467-019-14197-9 PMC6981116

[CR24] Hufnagel B, Soriano A, Taylor J, Divol F, Kroc M, Sanders H, Yeheyis L, Nelson M, Péret B (2021) Pangenome of white lupin provides insights into the diversity of the species. Plant Biotechnol J 19(12):2532–2543. 10.1111/pbi.13678 34346542 10.1111/pbi.13678 PMC8633493

[CR25] Juliana P, Singh RP, Singh PK, Crossa J, Huerta-Espino J, Lan C, Bhavani S, Rutkoski JE, Poland JA, Bergstrom GC, Sorrells ME (2017) Genomic and pedigree-based prediction for leaf, stem, and stripe rust resistance in wheat. Theor Appl Genet 130(7):1415–1430. 10.1007/s00122-017-2897-1 28393303 10.1007/s00122-017-2897-1 PMC5487692

[CR26] Kaler AS, Gillman JD, Beissinger T, Purcell LC (2020) Comparing different statistical models and multiple testing corrections for association mapping in soybean and maize. Front Plant Sci 10:1794. 10.3389/fpls.2019.01794 32158452 10.3389/fpls.2019.01794 PMC7052329

[CR27] Książkiewicz M, Nazzicari N, Yang H, Nelson MN, Renshaw D, Rychel S, Ferrari B, Carelli M, Tomaszewska M, Stawiński S, Naganowska B, Wolko B, Annicchiarico P (2017) A high-density consensus linkage map of white lupin highlights synteny with narrow-leafed lupin and provides markers tagging key agronomic traits. Sci Rep 7(1):15335. 10.1038/s41598-017-15625-w 29127429 10.1038/s41598-017-15625-w PMC5681670

[CR28] Lenth RV, Banfai B, Bolker B, Buerkner P, Giné-Vázquez I, Herve M, Jung M, Love J, Miguez F, Piaskowski J, Riebl H, Singmann H (2025) emmeans: Estimated Marginal Means, aka Least-Squares Means (Version 1.10.7) [Computer software]. https://cran.r-project.org/web/packages/emmeans/index.html

[CR29] Luo C, Bashir NH, Li Z, Liu C, Shi Y, Chu H (2024) Plant micrornas regulate the defense response against pathogens. Front Microbiol. 10.3389/fmicb.2024.1434798 39282567 10.3389/fmicb.2024.1434798 PMC11392801

[CR30] Meuwissen TH, Solberg TR, Shepherd R, Woolliams JA (2009) A fast algorithm for BayesB type of prediction of genome-wide estimates of genetic value. Genet Sel Evol 41(1):2. 10.1186/1297-9686-41-2 19284681 10.1186/1297-9686-41-2 PMC2637029

[CR31] Mierziak J, Wojtasik W (2024) Epigenetic weapons of plants against fungal pathogens. BMC Plant Biol 24(1):175. 10.1186/s12870-024-04829-8 38443788 10.1186/s12870-024-04829-8 PMC10916060

[CR32] Morgulis A, Coulouris G, Raytselis Y, Madden TL, Agarwala R, Schäffer AA (2008) Database indexing for production MegaBLAST searches. Bioinf (Oxford England) 24(16):1757–1764. 10.1093/bioinformatics/btn322 10.1093/bioinformatics/btn322 PMC2696921 18567917

[CR33] Patyi A, Kamp M, Arncken C, Biazzi E, Książkiewicz M, Messmer MM, Schneider M, Tava A, Lazzaro M (2025) Identification of a new QTL associated to reduced quinolizidine alkaloid content in white lupin (*Lupinus albus*, L.) and development of ultra-low alkaloid recombinants by stacking with the pauper allele. BMC Plant Biol 25(1):945. 10.1186/s12870-025-06951-7 40696276 10.1186/s12870-025-06951-7 PMC12281768

[CR34] Phan HTT, Ellwood SR, Adhikari K, Nelson MN, Oliver RP (2007) The first genetic and comparative map of white lupin (*Lupinus albus* L.): identification of QTLs for anthracnose resistance and flowering time, and a locus for alkaloid content. DNA Res 14(2):59–70. 10.1093/dnares/dsm009 17526914 10.1093/dnares/dsm009 PMC2779896

[CR35] Puthiyedth N, Zeinalinesaz F, Hou D, Zhang Y, Lin W, Yan Y (2025) Leveraging LASSO-based methodologies for enhanced SNP analysis in plant genomes. Bioinform Adv 5(1):vbaf014. 10.1093/bioadv/vbaf014 40092525 10.1093/bioadv/vbaf014 PMC11908641

[CR36] R Core Team (2022) R: A language and environment for statistical computing. [Computer software]. R Found Stat Comput. https://www.R-project.org/

[CR37] R Core Team (2024) R: A language and environment for statistical computing*.* [Computer software]. R Found Stat Comput. https://www.R-project.org/

[CR38] Rychel-Bielska S, Nazzicari N, Plewiński P, Bielski W, Annicchiarico P, Książkiewicz M (2020) Development of PCR-based markers and whole-genome selection model for anthracnose resistance in white lupin (*Lupinus albus* L.). J Appl Genet 61(4):531–545. 10.1007/s13353-020-00585-1 32968972 10.1007/s13353-020-00585-1 PMC7652745

[CR39] Sallam AH, Endelman JB, Jannink J-L, Smith KP (2015) Assessing Genomic Selection Prediction Accuracy in a Dynamic Barley Breeding Population. Plant Genome 8(1):plantgenome2014.05.0020. 10.3835/plantgenome2014.05.0020 10.3835/plantgenome2014.05.0020 33228279

[CR40] Schmidt P, Hartung J, Bennewitz J, Piepho H-P (2019) Heritability in plant breeding on a genotype-difference basis. Genetics 212(4):991–1008. 10.1534/genetics.119.302134 31248886 10.1534/genetics.119.302134 PMC6707473

[CR41] Schwertfirm G, Schneider M, Haase F, Riedel C, Lazzaro M, Ruge-Wehling B, Schweizer G (2024) Genome-wide association study revealed significant SNPs for anthracnose resistance, seed alkaloids and protein content in white lupin. Theor Appl Genet 137(7):155. 10.1007/s00122-024-04665-2 38858311 10.1007/s00122-024-04665-2 PMC11164739

[CR42] Sharma DK, Chatterjee M, Kaur G, Vavilala S (2022) 3—Deep learning applications for disease diagnosis. In D. Gupta, U. Kose, A. Khanna, & V. E. Balas (Eds.), Deep Learning for Medical Applications with Unique Data (pp. 31–51). Academic Press. 10.1016/B978-0-12-824145-5.00005-8

[CR43] Skøt L, Nay MM, Grieder C, Frey LA, Pégard M, Öhlund L, Amdahl H, Radovic J, Jaluvka L, Palmé A, Ruttink T, Lloyd D, Howarth CJ, Kölliker R (2024) Including marker x environment interactions improves genomic prediction in red clover (Trifolium pratense L.). Front Plant Sci. 10.3389/fpls.2024.1407609 38916032 10.3389/fpls.2024.1407609 PMC11194335

[CR44] Talhinhas P, Baroncelli R, Le Floch G (2016) Anthracnose of lupins caused by colletotrichum lupini: a recent disease and a successful worldwide pathogen. J Plant Pathol 98(1). 10.4454/JPP.V98I1.040

[CR45] Wang J, Zhang Z (2021) GAPIT version 3: boosting power and accuracy for genomic association and prediction. Genom Proteom Bioinform 19(4):629–640. 10.1016/j.gpb.2021.08.005 10.1016/j.gpb.2021.08.005 PMC9121400 34492338

[CR46] Wright S (1921) Correlation and causation. In J Agric Res 20(7):557. https://cir.nii.ac.jp/crid/1370567187556110595?lang=en

[CR47] Yang H, Lin R, Renshaw D, Li C, Adhikari K, Thomas G, Buirchell B, Sweetingham M, Yan G (2010) Development of sequence-specific PCR markers associated with a polygenic controlled trait for marker-assisted selection using a modified selective genotyping strategy: A case study on anthracnose disease resistance in white lupin (*Lupinus albus* L). Mol Breed 25(2):239–249. 10.1007/s11032-009-9325-4

[CR48] Yang X, Zhang L, Yang Y, Schmid M, Wang Y (2021) miRNA mediated regulation and interaction between plants and pathogens. Int J Mol Sci 22(6):2913. 10.3390/ijms22062913 33805611 10.3390/ijms22062913 PMC7999934

[CR49] Zhang Z, Schwartz S, Wagner L, Miller W (2000) A greedy algorithm for aligning DNA sequences. J Comput Biology: J Comput Mol Cell Biology 7(1–2):203–214. 10.1089/10665270050081478 10.1089/10665270050081478 10890397

[CR50] Zhang W, Boyle K, Brule-Babel A, Fedak G, Gao P, Djama Z, Polley B, Cuthbert R, Randhawa H, Graf R, Jiang F, Eudes F, Fobert P (2021) Evaluation of genomic prediction for Fusarium head blight resistance with a multi-parental population. Biology 10:756. 10.3390/biology10080756 34439988 10.3390/biology10080756 PMC8389552

[CR51] Zhao T, Wang F, Qi J, Chen Q, Zhu L, Liu L, Yan L, Chen Y, Yang C, Qin J (2025) Genome-wide association analysis study and genomic prediction for resistance to soybean mosaic virus in soybean population. BMC Plant Biol 25:837. 10.1186/s12870-025-06775-5 40604369 10.1186/s12870-025-06775-5 PMC12220144

